# Diagnostic performance of swept-source optical coherence tomography in the detection of tooth cracks: a narrative review

**DOI:** 10.2340/aos.v85.45800

**Published:** 2026-04-16

**Authors:** Anfal AlQussier

**Affiliations:** Department of Restorative Dental Science, College of Dentistry, Taibah University, Madinah, Saudi Arabia

**Keywords:** tomography, optical coherence, tooth fractures, diagnostic imaging, dental enamel, tooth crack

## Abstract

**Objectives:**

Tooth cracks are among the most common clinical findings that can affect the prognosis of the tooth. The swept-source optical coherence tomography (SS-OCT) is a non-invasive technique that was developed to identify cracks or fractures. Even though the diagnostic value of SS-OCT has been studied by several researchers, the available evidence is still inconclusive. This review aims to evaluate the reported performance of SS-OCT in detecting tooth cracks and to identify factors described in the literature that may affect it.

**Materials and methods:**

An electronic search was conducted on PubMed, Scopus, Web of Science, and Google Scholar to extract and review the English articles published between January 2012 and December 2024. The keywords were optical coherence tomography, swept-source OCT, SS-OCT, tooth crack, dental crack, enamel crack, dentin crack, and fracture detection. Studies published in English that investigated SS-OCT for the detection of enamel, dentin, or root cracks were included, whereas review articles, non-dental OCT studies, and studies focusing solely on other diagnostic methods were excluded.

**Results:**

The literature search identified 121 articles; 14 of them met the criteria and were included. Out of the 14 included articles, only one clinical article was included.

**Conclusions:**

The SS-OCT is a non-invasive and radiation-free imaging method for detecting tooth cracks, with higher diagnostic performance reported for enamel cracks. However, its performance in deeper dentin and root cracks appears to be affected by light penetration limitations and light scattering. Further technological development and well-designed clinical studies are needed to clarify its clinical utility.

## Introduction

In 1964, Cameron proposed the concept of cracked tooth symptoms, which was originally described as an incomplete tooth fracture [1]. According to the American Association of Endodontists, a cracked tooth can be characterized as ‘a thin surface disruption of enamel and dentin, and possibly cementum, of unknown depth or extension’ [2]. These clinical manifestations depend on where and how far the fracture is [3, 4].

Craze line is a crack in the tooth that is asymptomatic. It occurs on the enamel surface parallel to the prismatic orientation due to occlusal forces or thermocycling. The cracks may also spread to cause vertical root fractures [5–7]. It has been found that the incidence rate of cracked teeth in adults aged 30–50 is between 34 and 74%, and the risk is higher in females. The risk of having a cracked tooth is high among older people who have more retained teeth [3, 8]. The mandibular molars are more likely to have an enamel crack due to the wedging effect of the mesiopalatal cusp of the maxillary molars [3].

The prognosis of a crack is unpredictable, and the most appropriate treatment technique should be identified with the help of the proper diagnosis of the size and localization of the crack [5, 9]. The early detection of the existence of a crack can improve the prognosis of the tooth and reduce the growth of the crack. Sadly, the cracked tooth may be hard to diagnose since the symptoms vary based on the crack site and the extent of the crack [5].

Transillumination, visual inspection, bite tests, cold testing, and radiographs are the currently used diagnostic tools, which, however, do not always give enough information regarding the depth or orientation of the crack [7, 10, 11].

In order to increase the detection of cracks, optical coherence tomography (OCT) has been examined in a number of studies [5, 10–17]. OCT is a non-invasive, non-ionizing imaging technique that provides high-resolution cross-sectional images of biological tissues. It was first introduced by Huang et al. [18]. It is currently applied in ophthalmology, dermatology, and cardiology. In dentistry, it has also been used in the detection of caries, cracks, periodontal structures, and oral lesions [7, 19].

OCT operates in the same way as the ultrasonic pulse echo systems, but with the difference that it employs near-infrared light (wavelength of 850 and 1300 nm) in place of sound [4, 7]. Another variant of OCT is swept-source OCT (SS-OCT), which uses the Fourier domain technique [3, 20]. SS-OCT uses a tunable laser (centered around 1300 nm) that sweeps over a certain range to generate high-resolution cross-sectional images. The use of longer wavelengths enhances penetration depth and enables imaging depth up to 2.5 mm into enamel and dentin. High light penetration, along with axial and lateral image resolution enhancement (around 5–15 µm) and real-time imaging acquisition, allows visualization of enamel and dentin cracks [3, 20].

Clinically, SS-OCT offers real-time cross-sectional images without the harm of radiation exposure. These make it a useful tool for early diagnoses and monitoring of dental cracks [20].

Most of the available studies have been conducted under laboratory conditions, with only limited clinical application to assess the efficacy of SS-OCT in the detection of tooth cracks. The majority of the studies were on enamel, and fewer studies were on dentin and root surfaces [4, 7, 10, 13, 14, 21–24]. There is limited information on the effectiveness of SS-OCT in identifying the various kinds of tooth cracks and, particularly, the deep ones, such as those in dentin and root surfaces. Therefore, this review aims to evaluate the reported diagnostic performance of SS-OCT in detecting tooth cracks and to identify factors described in the literature that may influence this performance.

## Methods

It is a narrative review that has a structured literature search. A search of literature was performed in PubMed, Scopus, Web of Science, and Google Scholar regarding the included English-language articles published from January 2012 to December 2024. In PubMed, MeSH terms were combined with free-text keywords using Boolean operators to optimize the search sensitivity and specificity. The keywords were optical coherence tomography, swept-source OCT, SS-OCT, tooth crack, dental crack, enamel crack, dentin crack, and fracture detection.

### Inclusion and exclusion criteria

The inclusion criteria were articles that were published in English, investigated the use of SS-OCT in the detection of enamel, dentin, or root cracks were only considered. The exclusion criteria were that the review articles, non-dental OCT studies, and articles that concentrated only on other methods of diagnosis. The abstract screening and full-text screening of the included articles were carried out.

**Figure 1:**
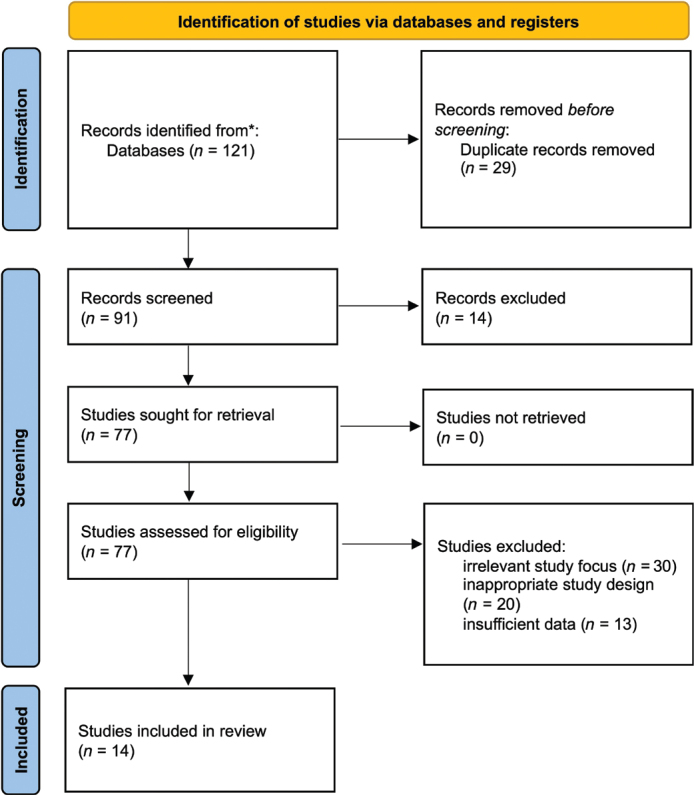
PRISMA flow diagram for studies retrieved through the searching and selection process.

The database search has revealed 121 articles. The number of records that were discarded before screening because of duplication was 29. After screening the titles and abstracts, 14 articles were eliminated. Sixty-three reports were excluded, 30 records were excluded due to irrelevant study focus, 20 were excluded due to inappropriate study design, and 13 were excluded due to insufficient data. A total of 14 studies that were in line with the inclusion criteria were used in this narrative review. Figure 1 shows the PRISMA flow diagram for studies retrieved through the searching and selection process.

The quality assessment has not been done in this review since it is just a summary and contextualization of the existing evidence, not an assessment of the risk of bias.

In particular, identifying cracks’ location and providing methodological details highlight the differences in OCT system specifications and imaging methods that could affect diagnostic results and offer a clinical context. In addition, disclosing the purpose, main results, and conclusions of each study enables a consistent comparison of the diagnostic precision and general effectiveness of SS-OCT in identifying cracks in tooth hard tissues.

## Results

Overall, the included studies reported a higher diagnostic performance of SS-OCT for detecting enamel cracks compared with deeper dentin and root cracks, which could be attributed to variations in optical behavior and imaging conditions. Only one study [8] included clinical assessment, while the other 13 included studies were *in vitro*. Table 1 shows a summary of each reviewed study, including the authors of the study, the article’s title, the aim of the study, the type of crack, and the methodologies. It also summarizes the main results and conclusions of each study, and how effective SS-OCT was in detecting enamel, dentin, or root cracks.

**Table 1:** Included studies, aim, type of crack, and methodology.

Study	Article’s title	Aim of study	Type of the study	Carack location	Methodology	Main results and conclusions
Imai et al., 2012 [5]	Noninvasive Cross-sectional Visualization of Enamel Cracks by Optical Coherence Tomography in vitro	To assess the diagnostic ability of SS-OCT for detecting enamel cracks	In vitro	Enamel	• Twenty extracted teeth were observed initially under transillumination, direct visual examination, and to record any cracks with a light intensity of 650 mW/cm^2^. • Seventy-one locations were selected randomly and observed under SS-OCT (of a central wavelength of 1330 nm) with the scanning beam oriented perpendicular to the surface. • The teeth were then sectioned under water coolant and examined under CLSM at 100x magnification to validate the observation of the SS-OCT images.	• The existence and extent of enamel fractures were observed using SS-OCT. • SS-OCT can also detect even enamel cracks beyond the dentin-enamel junction (DEJ). • The accuracy of the SS-OCT is superior to the conventional visual examination for enamel cracks, especially for whole-thickness enamel cracks. • SS-OCT observations are well correlated with the CLSM observations. • SS-OCT may provide stable and objective visual information since it showed superior inter-examiner reproducibility than the visual inspection method.
Nakajima et al., 2012 [27]	Noninvasive cross-sectional imaging of incomplete crown fractures (cracks) using swept-source optical coherence tomography	To investigate the ability of SS-OCT to detect cracks in extracted teeth	In vitro	Enamel	• Artificial cracks were created in 30 extracted intact porcine premolars by impacting the teeth occlusally with a steel rod plunging. • Teeth were examined under SS-OCT (Santec OCT-2000; Santec Co., Komaki, Japan) with a central wavelength of 1310 nm and a 20-kHz sweep rate. • To assess the effect of the scanning angle, SS-OCT focused light was projected from three directions at the same plane: buccal or lingual (90º), 45º to the mesial, and 45º to the distal surfaces. • The teeth were also evaluated under an optical stereomicroscope (SZH-ILLB; Olympus, Tokyo, Japan) at 10x magnification. • All teeth were assessed and photographed before and after impaction. • Five histological sections were prepared corresponding to images obtained from SS-OCT and examined under an optical microscope at a magnification of 40x for observation validation. • Images from SS-OCT and histological sections were analyzed using software (ImageJ version 1.42q; NIH, Bethesda, MD, USA) to measure cracks’ dimensions.	• Crack measurements obtained from SS-OCT were significantly correlated with the histological examinations. • SS-OCT was able to detect enamel cracks and lamellae as highlighted lines due to light scattering and provide a quantitative evaluation of the crack that could not be achieved by traditional examination methods. • They also reported that changing the scanning angle could affect the dimensions of the crack in the obtained images according to penetration depth.
Lee et al., 2016 [10]	Dental optical coherence tomography: a new potential diagnostic system for cracked-tooth syndrome	To assess the reliability and clinical effectiveness of OCT in detecting cracked teeth and to compare it with visual inspection, transillumination, and micro-CT.	In vitro	Enamel	• A total of 109 surfaces of 61 extracted teeth (12 incisors, 23 premolars, and 26 molars) were examined using different diagnostic methods. • Visual examination was done with the naked eye and was repeated under transillumination using a light-emitting diode (LED). • Teeth were also tested under SS-OCT and micro-CT • The reliability of SS-OCT was verified by comparing the number of detected crack lines with the number of cracks observed by other conventional methods: visual inspection, transillumination, and micro-CT.	• The authors found that the crack detection ability of the SS-OCT is superior to the visual examination with the naked eye. • Moreover, compared to micro-CT and trans-illumination, SS-OCT showed superior or similar detection ability. • Structural cracks and craze lines could be easily distinguished in images obtained by SS-OCT.
Segarra et al., 2017 [21]	Three-Dimensional Analysis of Enamel Crack Behavior Using Optical Coherence Tomography	To employ 3D SS-OCT to nondestructively analyze the behavior of enamel cracks in various regions of teeth	In vitro	Enamel	• Eighty extracted human teeth were examined using 3D SS-OCT for enamel crack patterns at different locations. Maxillary and mandibular incisors, canines, premolars, and molars (*n* = 10), which were involved in mastication, were observed at functional, non-functional contacting, and non-contacting locations. • Teeth were observed under SS-OCT (Yoshida Dental MFG, Tokyo, Japan) with a central wavelength of 1310 nm, a scan range of 140 nm, a horizontal resolution of 30 μm, and a depth resolution in the air of 11 μm. • The handheld scanning probe was oriented at 90° to the occlusal surface. • SS-OCT findings were validated using CLSM after samples were sectioned with an Isomet diamond saw with water coolant at a slow speed to prevent new crack formation.	• Cracks appeared as bright lines in images obtained by SS-OCT. • 3D SS-OCT and CLSM observations were correlated with each other. • Occluso-gingival (vertical) oriented cracks were shown more on non-contacting surfaces of anterior teeth and non-functional cusps of posterior teeth. While superficial horizontal cracks and hybrid cracks were found more on contacting surfaces of incisors, cusps of canines, and functional cusps of posterior teeth. • A strong correlation was reported for crack pattern, tooth type, and crack location on the tooth. • The authors suggested using 3D SS-OCT as a nondestructive method for enamel crack behavior analysis studies.
Tabata et al., 2017 [35]	Assessment of enamel cracks at adhesive cavosurface margin using three-dimensional swept-source optical coherence tomography	To assess enamel cracks at the cavosurface margins of resin composite restorations utilizing SS-OCT	In vitro	Enamel	• Cavities with enamel cavosurface were prepared on 60 bovine teeth at the middle or cervical thirds of each tooth. • Only half of the cavities (*n* = 30) were treated with phosphoric acid gel (Kuraray Noritake Dental Tokyo, Japan) before bonding application. • All cavities were restored using a two-step self-etch adhesive (Clearfil SE Bond, Kuraray Noritake Dental, Tokyo, Japan), and a flowable composite (Estelite Flow Quick, Shade A2, Tokuyama Dental, Tainai, Japan) was placed in one increment. • After 7 days in water at 37°C, SS-OCT with a centered wavelength at 1310 nm at a 50 kHz rate (Yoshida Dental MFG, Japan) was used to generate 3D and cross-sectional images of the enamel margins. Cracks at the enamel cavosurface were measured using a 5-point scale. • Five representative samples with enamel cracks were assessed using CLSM at an optical magnification of ×10 (3D Laser Scanning Confocal Microscope; Keyence, Osaka, Japan) after sectioning under running water for observation validation.	• 3D SS-OCT can detect cracks at the enamel-resin composite margin noninvasively. • More enamel cracks were found at the cervical margins compared to the enamel at the middle third. • Using phosphoric etching results in higher enamel crack formation compared to non-etched enamel.
Kim et al., 2017 [25]	Automatic detection of tooth cracks in optical coherence tomography images	To compare the crack visibility and image quality between SS-OCT and conventional diagnostic methods, and to develop an automatic tooth crack detection method using SS-OCT.	In vitro	Enamel	• Six teeth were artificially cracked using a steady external force. • Images of the cracked teeth were obtained using SS-OCT (Oz-tec Co., Daegu, Korea) with a central wavelength of 1310 nm and other conventional methods: transillumination, intraoral radiograph, and CBCT. • A software (MATLAB, MathWorks, Natick, MA, USA) was used to analyze the SS-OCT obtained images. • A Hough transform was employed to detect a straight line, and a local adaptive threshold was employed to enhance the contrast of the edges.	• SS-OCT’s ability to detect fine cracks is superior to or comparable to the transillumination method, which can not determine the depth of the crack. • Conventional diagnostic methods are not able to detect cracks with less than 100 μm, unlike SS-OCT, which can even detect craze lines. • Using the Hough transform, cracks can be automatically detected. • SS-OCT is not able to detect vertical root fractures.
Kang et al., 2017 [36]	Tooth cracks detection and gingival sulcus depth measurement using optical coherence tomography	To evaluate SS-OCT performance for the detection of tooth cracks and for measuring gingival sulcus depth.	In vitro	Enamel	• Artificial cracks were prepared using an external force of 350–500 N by moving the probe at a speed of 60 cm/s. • Teeth were examined using SS-OCT with a center wavelength of 1310 nm (Oz-tec Co., Daegu, Korea) • Images were analyzed by MATLAB software (Ver. R2016b, MathWorks, Natick, MA, USA). Images’ contrasts were improved using a local adaptive threshold, and the Hough transform was used for straight lines detection.	• The used SS-OCT can detect the location of tooth cracks that could not be visualized with the naked eye, and visualize the deep periodontal pockets automatically. • Image processing techniques are needed for accurate crack detection by enhancing image quality.
Ei et al., 2019 [12]	Three-dimensional assessment of proximal contact enamel using optical coherence tomography	To use 3D SS-OCT to evaluate the relationship between enamel microcracks and demineralization in premolar proximal contact areas	In vitro	Enamel	• Fifty extracted intact premolars were examined using the International Caries Detection and Assessment System (ICDAS) and SS-OCT (prototype, Yoshida Dental MFG, Tokyo, Japan) for microcracks and demineralization detection. • SS-OCT observations were confirmed using CLSM (VK-X150 series, Keyence, Osaka, Japan) after teeth sectioning with a low-speed Isomet diamond saw under water coolant.	• SS-OCT was able to show enamel microcracks as clear, bright lines within enamel that could not be seen by the naked eye. • Enamel microcracks in the proximal surfaces could act as a confounding factor for the caries process. • CLSM validated the microcrack pattern seen with SS-OCT images. • 3D SS-OCT is a valid, comprehensive diagnostic tool for enamel surface investigation.
Sahyoun et al., 2020 [37]	An Experimental Review of Optical Coherence Tomography Systems for Noninvasive Assessment of Hard Dental Tissues	To compare three commercial OCT systems with alternative wavelengths, imaging lenses, and detectors for enamel imaging	In vitro	Enamel	• The performance of two spectral-domain (SD) OCTs with different central wavelengths, (A) 840 nm [Lumedica, OQ LabScope 1.0] and (B) 1300 nm [Thorlabs, Tel320], and (C) a swept-source SS-OCT system [Thorlabs OCS1300SS] with a central wavelength of 1325 nm and optional polarization-sensitive (PS) detection was compared. The three systems were used to assess intact human enamel clinically and eroded bovine enamel samples in the laboratory. • High and low numerical aperture (NA) imaging lenses were used with SS-OCT.	• SD-OCT with a longer central wavelength (1300 nm) showed 2.6 times deeper imaging depth compared to SD-OCT with 840 nm. • A larger visual field could be achieved with low NA, while the use of higher NA results in higher contrast and higher lateral resolution of the images. • Polarization-sensitive imaging SS-OCT is employed to eradicate the birefringent banding artifacts that are visible in the OCT systems.
Wada et al., 2015 [8]	Clinical assessment of non-carious cervical lesions using swept-source optical coherence tomography	To study the mechanism of non-carious cervical lesions (NCCLs) using SS-OCT in vitro and in vivo	Combined (in vitro and clinical)	Dentin	Two-part study: • In the in vitro part, the attenuation coefficient (μt) of the dentin from SS-OCT signals at NCCl was compared to the mineral loss measured by transverse microradiography to establish a μt threshold. That could be used clinically to differentiate the demineralization of cervical dentin. • In the clinical part, 242 buccal surfaces were examined using SS-OCT to determine the degree of dentin demineralization, the presence of cracks, and the dimensions of NCCLs.	• Dentin demineralization was found in most examined NCCls and was associated with larger NCCLs. • SS-OCT without polarized sensitivity (PS) results in superior detection ability of tooth cracks. SS-OCT can detect cervical cracks at DEJ.
Hovander et al., 2022 [14]	Optical coherence tomography evaluation of deep dentin crack removal techniques	To noninvasively evaluate the effectiveness of the deep coronal dentin crack removal technique using an SS-OCT	In vitro	Dentin	• Standard cracks were created in the pulpal floor of 40 intact extracted posterior teeth. • Samples were divided into five groups based on the crack removal technique using airborne-particle abrasion or burs; fissure, small round, medium round, or tapered fine diamond. • SS-OCT (Yoshida Dental) scanning was performed before and after crack removal, and three-dimensional image registration was used to analyze the results.	• Among the treatment groups, using particle abrasion results in the smallest crack propagation and the largest amount of dentin removal. All burs lead to crack formation with no significant difference in crack dimensions. The volume of dentin removed depends on the size of the bur used. • SS-OCT was able to detect dentin microcracks of decoronated posterior teeth.
Luong et al., 2018 [38]	Cross-sectional imaging of tooth bonding interface after thermal stresses and mechanical fracture	To assess the impact of thermocycling and flowable resin composite on micro-tensile bond strength (MTBS), crack formation, and mechanical properties of the bonding interface using SS-OCT and nanoindentation	In vitro	Dentin	• The study used MTBS beams made from human dentin and hybrid composite with/without flowable lining. Only half of the samples were subjected to 10,000 thermocycles. • 2D images were obtained by SS-OCT to detect cracks before and after the MTBS test. Hardness was evaluated using a nanoindentation test. • For validation, samples were observed under CLSM (CLSM-1LM21H/W, Lasertec, Yokohama, Japan) at ×250 and ×500 magnification levels.	• MTBS was significantly higher in samples restored with flowable lining • Thermocycling significantly affects the hardness of dentin in the resin composite. However, no significant effect of thermocycling on crack percentages in dentin. • SS-OCT can disclose cracks in both resin composite restorations and dentin. Cracks in dentin showed as bright dots and short lines under the surface. • SS-OCT can detect and evaluate the cracks at the bonding interface noninvasively.
Oliveira et al., 2017 [7]	Detection of Apical Root Cracks Using Spectral Domain and Swept-source Optical Coherence Tomography	To evaluate the efficacy of two OCT devices in detecting apical dentinal microcracks.	In vitro	Root	• Root canal preparations were done for 20 extracted mandibular incisors using R40 Reciproc files (VDW, Munich, Germany). • Teeth were examined using micro-CT (XTH225ST; Nikon, Tokyo, Japan) to determine the presence of cracks. The X-ray beam was projected perpendicularly to the long axis of the tooth. • Teeth were also scanned using spectral domain OCT (SD-OCT) (Callisto, Thorlabs, Newton, NJ) with a central wavelength of 930 nm and SS-OCT systems (OCS1300SS, Thorlabs) with a central wavelength of 1325 nm. The light beams of both OCTs were projected parallel to the long axis of the teeth.	• Both OCTs showed a reliable detection ability, making them highly promising tools for detecting apical dentinal microcracks. • SD-OCT presents a slightly superior but not statistically significant – diagnostic performance compared to SS-OCT. Both systems showed high intra-examiner agreement. • Cracks were presented as clefts separating the dentin in both evaluated OCTs • OCT with a longer wavelength allows deeper visualization, but with lower image resolution
Rashed et al., 2019 [17]	Evaluation of Crack Formation and Propagation with Ultrasonic Root-End Preparation and Obturation Using a Digital Microscope and Optical Coherence Tomography	To investigate the impact of apicoectomy, ultrasonic root-end preparation, and root-end filling on the occurrence and spread of cracks using a digital microscope (DM) and SS-OCT Also, to evaluate the effectiveness of SS-OCT in crack detection by comparing it to micro-CT as a reference standard.	In vitro	Root	• Lower incisors (*n* = 30) were sectioned 2 mm above the cementoenamel junction using a low-speed Isomet saw under water coolant. • Teeth were endodontically treated and then underwent apicoectomy and ultrasonic root apex cavity preparation. • Roots were then filled with mineral trioxide aggregate (MTA) or super-EBA or left unfilled (*n* = 10). • Teeth were examined immediately after apicoectomy, ultrasonic root-end preparation, and root-end filling, and repeated after 2 weeks, a month, and 2 months. • Examinations were done using DM (DM; VH-8000, Keyence, Osaka, Japan) with a magnification of 40x with an external LED light source. SS-OCT (Santec OCT-2000, Santec Co., Komaki, Japan) scanning was done at the same periods with the laser beam projected parallel to the long axis of the tooth. • The Micro-CT (inspeXio SMX-100CT, Shimadzu, Kyoto, Japan) examinations were done before treatment and after 2 months for validation.	• Both apicoectomy and ultrasonic preparation result in dentinal crack formation. • A very weak correlation was reported between observations obtained by SS-OCT and DM. DM has significantly superior sensitivity in detecting dentinal cracks compared with SS-OCT. • The low performance of SS-OCT could be due to the optical properties of dentin. • Cracks were presented in SS-OCT images as white lines.

SS-OCT: swept-source optical coherence tomography; CLSM: confocal laser scanning microscopy; CBCT: cone beam computed tomography; micro-CT: micro-computed tomography.

All this information gives an overview of the available research on the diagnostic accuracy of SS-OCT in detecting different types of tooth cracks.

## Discussion

### Diagnostic methods and limitations

Various diagnostic methods are used clinically and in laboratories for tooth crack detection. Transillumination, for example, is a simple chairside method in which light passes through the tooth structure until a discontinuity, such as a crack or caries, alters its path. When a crack is present, the sudden change in refractive index between tooth structure and the crack space occurs, and a bright–dark boundary appears [25].

While transillumination is able to confirm the presence of a crack, it cannot differentiate between crack types or determine their depth, extent, or orientation. This limits its diagnostic value to a binary detection outcome [25].

Radiographic modalities – including intraoral radiography, cone beam computed tomography (CBCT), and micro-computed tomography (micro-CT) – have also been used to identify structural defects and cracks. Nevertheless, their spatial resolution typically approaches 100 µm, which is insufficient for detecting early micro-cracks or subtle enamel discontinuities during the initial stages of progression [11, 25]. This diagnostic limitation highlights the need for advanced imaging methods capable of obtaining high-resolution, depth-resolved information without relying on ionizing radiation.

Traditional diagnostic methods are essential for identifying advanced or obvious cracks, but their poor resolution and limited ability to characterize subtle structural changes highlight the need for higher-resolution, non-invasive imaging systems – an essential rationale for exploring OCT/SS-OCT technologies in crack detection.

### Benefits of SS-OCT in crack detection

SS-OCT has gained increasing attention as a diagnostic tool for tooth cracks as it provides high-resolution, cross-sectional imaging without the need for invasive sectioning. As a real-time, non-destructive modality, OCT can generate 2D and 3D images that reveal subtle structural discontinuities that remain undetectable using conventional methods. In accordance with this, Dao Luong et al. [26] showed that SS-OCT can clearly visualize interfacial and dentin cracks after a microtensile test, emphasizing its sensitivity to subtle structural defects. SS-OCT further extends these advantages by offering superior resolution and deeper penetration compared with CBCT and intraoral radiography, while also eliminating exposure to ionizing radiation. This highlights its potential advantage in situations where radiation exposure is a concern as in pregnant women and children. The compact size of SS-OCT equipment also enhances its usability compared with bulkier radiographic equipment [25, 27].

The light scattering properties, composition, and refractive indices (*n*) of scanned structures are the foundations of OCT’s diagnostic ability to distinguish between various tooth structures and crack space [5]. The light penetration depth of OCT is highly affected by the translucency of the scanned medium. Because sound enamel structure is almost transparent at the wavelength range used in OCT systems, even small disturbances in its optical pathway become visible as noticeable shifts in signal intensity [28]. OCT images show both enamel and dentin as white structures, which are distinguished from each other by a dark line representing the dentinoenamel junction (DEJ) [22]. Swept-source OCT is an improved variant of OCT that uses a Fourier-domain acquisition principle, offering greater sensitivity than traditional time-domain systems. It employs an interferometer coupled with a narrow-linewidth, frequency-swept laser centered at a 1300-nm wavelength, while a detector records the interference signal over time. Modern SS-OCT devices are capable of producing cross-sectional images with near-microscopic resolution [22]. Notably, SS-OCT at 1300 nm has been reported to outperform visible-light transillumination at 473 nm in detecting fine and deep enamel cracks [5, 29].

While SS-OCT demonstrates strong potential for detecting enamel cracks, it is important to recognize that different OCTs vary in their suitability for this purpose. For example, polarization-sensitive OCT (PS-OCT) has been effectively used to reduce surface reflection, making it an effective tool for measuring lesion depth in enamel and dentin during demineralization studies. However, crack detection relies heavily on the refractive index differences between air-/fluid-filled cracks and the surrounding tooth structures. Because PS-OCT minimizes this optical contrast, it is not considered proper for identifying tooth cracks [8].

Collectively, these remarks demonstrate how the underlying optical principles, imaging characteristics, and technical variations among OCT systems shape their effectiveness in crack diagnostics. They also reinforce the particular suitability of SS-OCT for non-invasive crack assessment and clarify the circumstances in which other modalities, such as PS-OCT, may be less appropriate for this purpose.

### Image formation and optical properties

For crack detection, OCT depends on the reflection phenomenon in which the light is refracted and partially reflected when it crosses through two different media with different *n* values. The crack space is assumed to be filled with air and water with *n* values of 1.0 and 1.33, respectively. On the other hand, the *n* value of the enamel is 1.631 ± 0.007 [30, 31]. Consequently, the presence of a crack in enamel results in a peak in the signal intensity profile of the backscattered light, which would be visible as a bright cluster in a 2D B-scan image [5, 32]. Many studies reported high performance of the SS-OCT in the detection of different types of enamel cracks, starting from craze lines to whole enamel thickness cracks, and determining their exact location and dimensions (Table 1). The higher sensitivity of SS-OCT for detecting enamel cracks is due to the penetration of the SS-OCT light to the whole thickness of the enamel [33].

As the light extends beyond enamel into the deeper dentin, the optical behavior becomes more complex. Like enamel, the *n* value of dentin is 1.4, which is different from that of the air and water. This makes the cracks visible as bright lines under SS-OCT [30]. However, dentin is composed of 50 vol% inorganic and 30 vol% organic (mostly type 1 collagen) compositions, and 20 vol% fluid. Due to its composition, dentin is optically non-linear and scatters light. As a result of its higher attenuation compared to the enamel, the light penetration depth within the dentin is smaller [22, 34]. In addition, the orientation of the dentinal structure affects the refractive index and light scattering pattern [34]. Thus, detection of deep dentin and root cracks could be affected and may require teeth sectioning, which is not applicable clinically, especially in teeth with full-thickness enamel. Still, SS-OCT helps in following crack extension by repeating scans of the same area over time, but deeper extension into dentin or roots is harder to assess because of scattering and limited penetration.

Overall, these optical characteristics clarify why SS-OCT provides reliable visualization of enamel cracks but remains limited in detecting deeper dentin or root defects, highlighting the need for further improvements in penetration and image clarity.

### Technical challenges and limitations of SS-OCT

Although the SS-OCT system shows a promising performance, it also demonstrates some limitations that limit its clinical application [13]. Its main limitation is its shallow light penetration depth (about 3 mm). This makes its ability to detect cracks in deeper structures, such as cracks in roots and deep dentin, limited clinically [5, 10, 25]. Research on dentin crack detection reported the ability of SS-OCT to detect cracks in dentin (Table 1). However, the methodology used in these studies used decoronated teeth [14] or dentin samples [38], which do not represent full clinical conditions. While Wada et al. [8] examined the cervical regions of the teeth with non-carious cervical lesions (NCCLs), where enamel thickness is minimal, making crack visualization easier.

Light penetration depth, image resolution, contrast, and field of view can be influenced by system components such as the light source, imaging lenses, filters, and detection units [17, 37]. Using a longer SS-OCT wavelength (1300 nm) allows deeper light penetration compared to traditional OCT systems [37, 39]. However, this improvement comes at the cost of reduced axial resolution, which may lead to less detailed images and reduced diagnostic performance [40]. Rashed et al. [17] reported lower sensitivity and specificity of SS-OCT for detecting root cracks compared with a digital microscope (DM), largely due to lower image resolution and blurred structures. To improve image quality and reduce noise, filters such as Gaussian and median filters have been recommended [17, 25, 36], whereas averaging filters may result in loss of fine structural details and further image blurring [25]. Therefore, selecting an appropriate filter is essential to optimize crack detection.

Utilizing a high numerical aperture (NA) offers better lateral resolution; however, it reduces the field of view, making the system more sensitive to small patient movements and limiting its clinical practicality. For routine clinical use, lower NA settings are generally more suitable because they provide a wider field of view and allow coverage of a larger surface area, although at lower magnification [37].

Together, these technical limitations help to explain why SS-OCT tends to work better in controlled laboratory conditions than in real clinical settings, especially when it comes to detecting deeper cracks. This highlights the need for continued improvements in light penetration, image resolution, and overall system stability before the technique can be used more reliably in practice.

### Scanning conditions and patient-related factors

Light penetration is also affected by other factors related to the scanned object, such as the presence of pellicle, surface roughness, and inclinations. Flat and smooth surfaces exhibit better light penetration depth for SS-OCT compared to occlusal irregular surfaces [40–42]. In addition, surface dehydration affects the performance of the SS-OCT, since enamel hydration significantly influences its transparency at 1300 nm wavelength [30, 43]. Hydrated enamel samples show lower surface reflection of the light and decreased light scattering [43], so it is critical to maintain the teeth hydrated during examination.

Since SS-OCT is a non-contact technique, patients usually do not report discomfort during examination [36]. However, due to its limited scanning range, multiple scans are needed to cover the area of interest, which makes the examination time longer and may create a higher chance of motion-related artifacts [13, 40]. One practical approach to overcome this is to perform a quick preliminary scan using near-infrared imaging to locate the suspected crack before carrying out a more detailed scan of the selected area [44].

In addition, the location of the examined tooth in relation to the SS-OCT probe could affect the performance and quantitative measurements of SS-OCT. It is sometimes difficult to achieve a perpendicular orientation of the probe in the mouth [27, 36]. Therefore, taking multiple images at different angles of the area of interest is suggested. Beyond these optical considerations, the relatively high cost of SS-OCT devices – compared with other diagnostic tools – also limits their widespread clinical use [13, 24]. Sahyoun et al. [37] compared two OCT systems with different costs and found that the low-cost OCT still provided adequate visualization of the first 500 μm with fast focusing. This suggests that choosing an OCT device in clinical practice may depend more on the specific diagnostic need rather than simply selecting the most advanced system available.

Overall, these factors make SS-OCT work well in controlled laboratory studies, but they also show why using it routinely in the clinic is still challenging. This highlights the need for better probe access, easier handling, and more affordable devices in the future.

### Future improvements and design innovations

To enhance the performance and handling of OCT, different studies were conducted to improve the design and size of OCT devices in different fields. Hu et al. [45], in 2024, developed a novel micro intraocular OCT probe employing a piezoelectric tube with quartered electrodes to create Lissajous scanning movements at the end of a single-mode fiber. The probe size could be altered from 2.41 to 0.51 mm based on the need [45]. Another advancement of OCT probes was reported by Kim et al. [46] in 2024. They introduced a portable 3D high-resolution dental OCT probe with a design like a commonly used intra-oral scanner. It can generate 3D images and quantitatively analyze cross-sectional images [46]. These developments suggest that further modifications in probe design may enhance imaging capabilities in deeper areas, such as the root canal or the gingival sulcus. In addition, Abu Saleah et al. [23] conducted a recent study in 2024 to enhance the ability of OCTs in measuring crack dimensions in 3D images using developed algorithms. Such a system enables better evaluation and further follow-up of the condition [23].

As these developments move closer to clinical use, some practical points during scanning should also be considered. During the use of SS-OCT, it is recommended to select an outer reference point to ease locating the area of interest for reevaluation and reducing errors. At the same time, practical steps taken during scanning can also influence the OCT signal and image quality. The use of a plastic cover on the handheld probe could have an impact on light reflection; therefore, other methods of infection control should be employed [47].

Overall, these recent improvements show that OCT technology is progressing; however, many of the new probe designs and software tools are still not fully adapted for routine dental use. This highlights the need for more practical design and better image-processing methods to support reliable clinical detection of deeper cracks using SS-OCT.

As a final note in this discussion, this review has some limitations. It is a narrative review, so a formal risk-of-bias assessment and meta-analysis were not performed. Also, most of the included studies were laboratory-based (*in vitro*), and only one included study involved a clinical assessment. In addition, only English articles were included, so relevant studies published in other languages may have been missed. Finally, the included studies were heterogeneous in terms of tooth types, crack definitions, study designs, and SS-OCT settings, which makes direct comparison among studies difficult. Therefore, the findings of this review should be taken with caution, and more well-designed clinical studies are still needed.

## Conclusions

This review highlights both the diagnostic strengths and current limitations of SS-OCT as a tooth crack diagnostic tool, while also pointing to factors that may influence its reported performance. SS-OCT offers high-resolution, radiation-free imaging, which is particularly effective for visualizing enamel cracks. However, its diagnostic performance decreases in deeper tissues due to limited light penetration, optical scattering, and practical challenges related to scanning conditions.

Even though recent innovations in probe miniaturization, imaging optics, and computational enhancement may improve future clinical performance, current evidence does not yet support its regular use for dentin/root crack detection. Future technological development, cost-effective system optimization, and well-designed clinical studies are essential to determine the full potential of SS-OCT as an adjunctive diagnostic modality in dentistry.

## Data Availability

Not applicable.
